# Mitigation of Black Streak Defects in AISI 304 Stainless Steel via Numerical Simulation and Reverse Optimization Algorithm

**DOI:** 10.3390/ma18143414

**Published:** 2025-07-21

**Authors:** Xuexia Song, Xiaocan Zhong, Wanlin Wang, Kun Dou

**Affiliations:** School of Metallurgy and Environment, Central South University, Changsha 410083, China

**Keywords:** steel, surface defect, reverse optimization, numerical simulation, continuous casting

## Abstract

The formation mechanism of black streak defects in hot-rolled steel sheets was investigated to address the influence of the process parameters on the surface quality during the production of 304 stainless steels. Macro-/microstructural characterization revealed that the defect regions contained necessary mold slag components (Ca, Si, Al, Mg, Na, K) which originated from the initial stage of solidification in the mold region of the continuous casting process, indicating obvious slag entrapment during continuous casting. On this basis, a three-dimensional coupled finite-element model for the molten steel flow–thermal characteristics was established to evaluate the effects of typical casting parameters using the determination of the critical slag entrapment velocity as the criterion. Numerical simulations demonstrated that the maximum surface velocity improved from 0.29 m/s to 0.37 m/s with a casting speed increasing from 1.0 m/min to 1.2 m/min, which intensified the meniscus turbulence. However, the increase in the port angle and the depth of the submerged entry nozzle (SEN) effectively reduced the maximum surface velocity to 0.238 m/s and 0.243 m/s, respectively, with a simultaneous improvement in the slag–steel interface temperature. Through MATLAB (version 2023b)-based reverse optimization combined with critical velocity analysis, the optimal mold slag properties were determined to be 2800 kg/m^3^ for the density, 4.756 × 10^−6^ m^2^/s for the kinematic viscosity, and 0.01 N/m for the interfacial tension. This systematic approach provides theoretical guidance for process optimization and slag design enhancement in industrial production.

## 1. Introduction

304 austenitic stainless steel is widely applied in elevator decoration, kitchenware, medical devices, chemical industries, and architectural decoration due to its favorable machinability, weldability, and superior corrosion resistance [1,2,3,4]. These applications demand stringent surface quality requirements. However, steel enterprises encounter surface defects including black streaks, interlayer scratches, and scarring during stainless steel production, with black streak defects representing the most severe and prevalent issue [5,6]. Such defects compromise the surface integrity, reduce products’ grades, and ultimately diminish enterprises’ competitiveness. The primary mechanism for black streak formation involves the entrapment of mold slag inclusions within the solidified shell during continuous casting [7]. Addressing these defects at their source constitutes a critical challenge in 304 stainless steel manufacturing processes to enhance the product quality and production efficiency.

Numerous studies have investigated mold flow fields and slag entrainment mechanisms in continuous casting processes [8,9,10,11]. Li et al. established a two-dimensional longitudinal numerical model of the flow–heat transfer in liquid mold slag along the steel surface, analyzing the influence of the shear velocity on the average longitudinal flow velocity near submerged entry nozzles to provide theoretical guidance for industrial practice [12]. Zheng et al. systematically examined the effects of the mold oscillation parameters on the flow patterns and electromagnetic stirring’s impacts on the multiphase flow and slag entrapment behaviors [13]. Marcin et al. developed a physical model considering the mold slag layer characteristics and validated their findings through water modeling experiments [8]. Wu et al. investigated the effects of the argon bottom-blowing parameters on slag entrainment using similar water modeling approaches. However, these studies primarily focused on theoretical aspects without sufficient industrial validation [14]. Critical limitations persist in quantifying slag entrainment probabilities and determining critical slag entrainment velocities—essential parameters for operational optimization in practical casting processes.

With the continuous increase in casting speeds, expansion of continuous casting steel grades, and higher quality requirements for cast strands, mold slag has become a key technology in continuous casting development [15,16,17,18]. For specific steel grades, particularly alloy steels and stainless steels, optimizing mold slag’s functionality under varying casting conditions is critical to ensure smooth production and high-quality strands. Mold slag fulfills essential metallurgical roles: protecting molten steel from atmospheric oxidation, minimizing heat loss, assimilating nonmetallic inclusions, lubricating the mold walls and shell, and regulating the heat transfer rate/uniformity [18]. Consequently, optimizing the mold slag properties holds significant implications for enhancing the strand quality. Yang et al. developed a shear-thinning mold slag design that reduces slag entrapment while maintaining the lubrication performance [19]. Wang et al. proposed a machine learning approach and conducted high-temperature experiments to predict the mold slag wetting angles, addressing the time-consuming nature of making direct measurements [20]. Ji et al. employed an optimized XGBoost model to predict the slag entrapment probabilities and conducted feature analysis using the Gini coefficient and SHAP values for flux optimization [21]. However, these studies were constrained by their use of limited mold slag variants and lacked precise optimization based on comprehensive performance parameters, highlighting the need for systematic, parameter-driven flux design frameworks.

This study addressed the black streak defects in the stainless steel products of a steel plant through comprehensive metallurgical analysis. Initial macro-/microstructural characterization via metallographic microscopy identified defect morphologies. Subsequent Scanning Electron Microscope (SEM) and Energy Dispersive X-ray Spectroscopy (EDS) revealed mold slag components within defect regions, confirming slag entrainment during continuous casting to be the root cause of the surface quality deterioration. To mitigate this issue, a three-dimensional coupled finite-element model for flow–heat transfer was developed to simulate the mold flow patterns under various process conditions. The model systematically evaluated the impacts of the casting speeds, the port angle, and the depth on both the molten steel flow velocity and slag–steel interface temperature distributions. Using MATLAB-based reverse optimization algorithms, key mold slag parameters were inversely determined by minimizing the maximum surface velocity. This parameter-driven optimization framework provides theoretical guidance for mold slag design, demonstrating significant potential for defect reduction and the provision of operational cost savings while advancing metallurgical process optimization methodologies.

## 2. Experiment Procedure and Mathematical Model

### 2.1. Experiment Method

**Figure 1:**
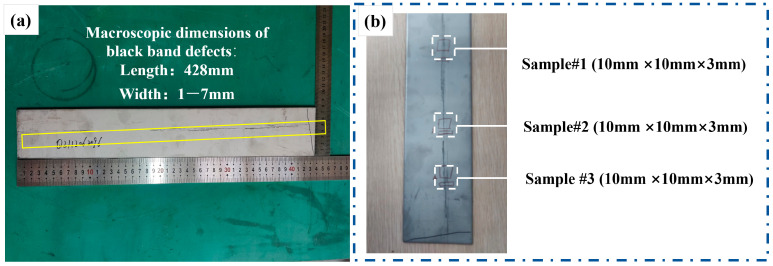
(**a**) Macroscopic dimensions of black streak defects; (**b**) sampling positions for microscopic and EDS analysis.

The main chemical composition of AISI 304 stainless steel (Castle Metals, Oak Brook, IL, USA) is shown in Table 1. Firstly, hot-rolled steel sheets containing visible dark band defects were subjected to macroscopic analysis, as shown in Figure 1a. For the macroscopic and microstructural investigation, representative specimens (10 mm × 10 mm × 3 mm) were extracted from the defective white-hot-rolled sheet using wire electrode cutting, following the sampling schematic shown in Figure 1b. Three specimens were sectioned along the rolling direction from the upper, middle, and lower regions of the defect-containing area. Secondly, the as-cut specimens were initially examined using an optical microscope (OLYMPUS, Tokyo, Japan) without surface preparation to document their macroscopic features. Subsequently, sequential preparation was required for microscopic analysis: the specimens were gently ground to achieve the partial removal of the thin defective layer and chemically etched in a 20% HCl (Sinopharm Group, Shanghai, China) solution for approximately 10 s, then thoroughly rinsed with anhydrous ethanol (Sinopharm Group, Shanghai, China) and dried with compressed air before re-examination under the optical microscope.

Additionally, for compositional analysis, the characterized specimens were transferred to a field emission scanning electron microscope equipped with energy-dispersive spectroscopy (SEM and EDS) equipment. And the SEM and EDS equipment were provided by Hitachi, Tokyo, Japan. Multiple spot analyses were performed on the defect zones to determine the elemental distributions and identify the potential causes of the black streaks’ formation.

### 2.2. Mathematic Method

**Figure 2:**
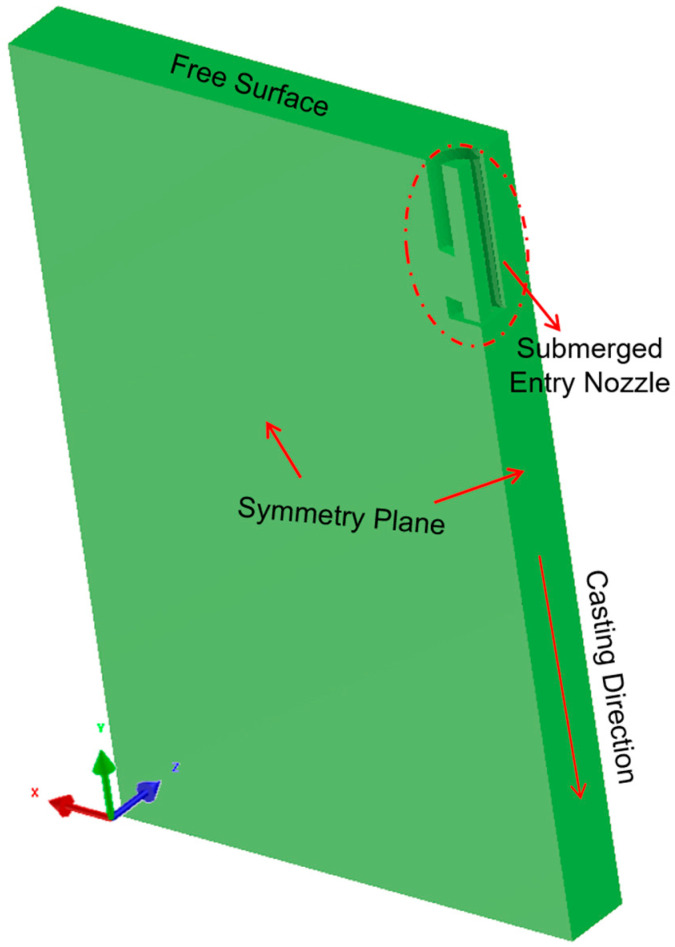
The CAD model of the mold used in this work.

A three-dimensional computational model was developed to investigate the effects of the continuous casting process parameters on the flow field, temperature distribution, and free surface velocity of molten steel. Based on the simulation results, the process parameters were optimized to minimize defect formation. A quarter-symmetry CAD model (Figure 2) was adopted to reduce the computational effort [22].

The following assumptions were implemented:

(I) The complex heat transfer at the curved mold meniscus was not subjected to any additional special treatments.

(II) The effects of mold oscillation, the curvature, and electromagnetic stirring on the melt flow and heat transfer were considered by incorporating the equivalent thermal conductivity and heat transfer coefficient.

The three-dimensional incompressible viscous flow of molten steel within the mold was governed by the fundamental conservation laws of mass, momentum, and energy. The mathematical framework comprised the continuity equation, momentum equation, energy equation, and κ−ε  quation, as shown in Equations (1)–(5) [23,24,25]. The specific formulations are expressed as follows:

(1) $$\frac{\partial \rho }{\partial t}+\nabla \cdot (\rho u)=0$$

(2) $$\rho \frac{\partial (u_{i}u_{j})}{\partial x_{j}}=-\frac{\partial p}{\partial x_{i}}+\frac{\partial }{\partial x_{j}}\left(\mu_{eff}\left(\frac{\partial u_{i}}{\partial x_{j}}+\frac{\partial u_{j}}{\partial x_{i}}\right)\right)+\rho g$$

(3) $$\frac{\partial (\rho h)}{\partial t}+\nabla \cdot (\rho uh)=\nabla \cdot \{(\kappa +\kappa_{t})\nabla \}-L_{f}\left[\frac{\partial (\rho f_{l})}{\partial t}+\nabla \cdot (\rho uf_{l})\right]$$

(4) $$\rho \frac{\partial \left(u_{j}K\right)}{\partial x_{j}}=\frac{\partial }{\partial }\left(\frac{\mu_{eff}}{\sigma_{K}}\cdot \frac{\partial K}{\partial x_{j}}\right)+G-\rho$$

(5) $$\rho \frac{\partial (u_{j}\varepsilon )}{\partial x_{j}}=\frac{\partial }{\partial x_{j}}(\frac{\mu_{eff}}{\sigma_{\varepsilon }}\cdot \frac{\partial \varepsilon }{\partial x_{j}})+\frac{\left(C_{1}G_{\varepsilon }-C_{2}\rho \varepsilon^{2}\right)}{K}$$

where *ρ* is the melt density, kg/m^3^, and u is the melt velocity, m/s. $u_{j}$ is the velocity in the *j* direction, m/s, and $x_{j}$ is the distance in the *j* direction, m. $u_{i}$ is the velocity in the *i* direction, m/s, and $x_{i}$ is the distance in the i direction, m. The pressure, Pa, is expressed as *P*, and the effective viscosity coefficient, kg/(m·s), is expressed as $\mu_{eff}$. The enthalpy is expressed as *h*, kJ/mol; the thermal conductivity and turbulent thermal conductivity are expressed as $\kappa \;and\;\kappa_{t}$, W/(m·°C); the node’s temperature is expressed as *T*; the latent heat of fusion is expressed as $L_{f}$, J/Kg; and the volume fraction of the liquid is expressed as $f_{l}$. The turbulent kinetic energy is expressed as *K*, J; ε is the dissipation rate of the turbulent kinetic energy, W/m^3^; *C_1_* and *C_2_* are empirical constants with values of 1.44 and 1.92; and $\sigma_{K}$ and $\sigma_{\varepsilon }$ are empirical constants with values of 1.0 and 1.3.

**Figure 3:**
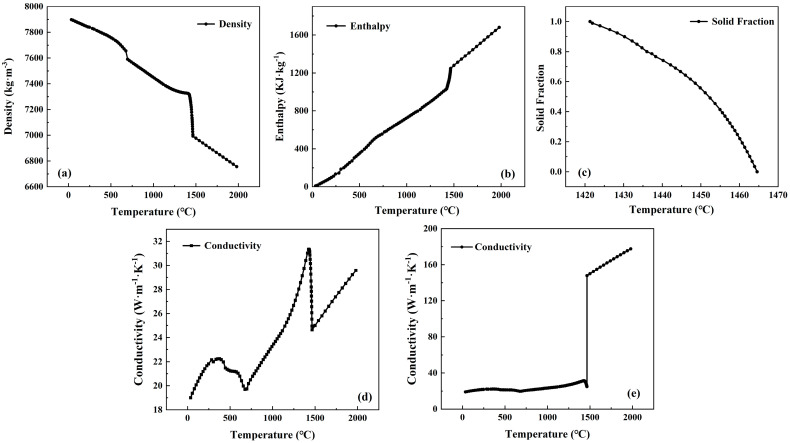
Temperature-dependent thermo-physical properties of 304 stainless steel. (**a**) Density; (**b**) enthalpy; (**c**) solid fraction (**d**) conductivity without melt convection; (**e**) conductivity with forced convection.

The temperature-dependent thermo-physical properties were calculated based on the Fe database in the software (2018) used [26], including the density, enthalpy, solid fraction, and thermal conductivity, which are shown in Figure 3. To further account for the effect of mold oscillation, the curvature, and electromagnetic stirring on the melt flow and heat transfer, the thermal conductivity above the liquidus temperature (1464 °C) was 5 times higher than the original values, as in previous work [27,28,29].

As for the flow and heat transfer boundary conditions, the following aspects were mainly considered.

(1) **Inlet boundary**: The velocity inlet was defined, and it was calculated based on the principle of mass conservation using conversion factors including the casting speed, nozzle dimensions, and mold dimensions. The turbulent kinetic energy and turbulent kinetic energy dissipation rate were determined using Equations (6) and (7), respectively. As for the heat transfer, the molten steel’s temperature at the inlet was set to the casting temperature.

(6) $$k=0.01\nu_{inlet}^{2}$$

(7) $$\varepsilon =\frac{2k^{1.5}}{d_{nozzle}}$$

where $\nu_{inlet}$ denotes the velocity of the inlet, m/s; $d_{nozzle}$ is the symbol for the nozzle’s hydraulic diameter, m.

(2) **Symmetry plane**: The velocity component perpendicular to the symmetry plane and the gradients of all the physical quantities along the normal direction of the symmetry plane were 0.

(3) **Wall boundaries**: Both the mold and nozzle walls were treated as no-slip solid walls. The normal velocity component and normal gradients of the other physical quantities at the wall surface were zero. Near-wall flow fields were resolved using standard wall functions. The average heat flux on both the narrow face and wide face of the mold was set to 2 MW/m^2^.

(4) **Outlet**: The velocity at the lower outlet of the mold was defined as the casting speed.

The necessary simulation parameters used in this modeling work are summarized and listed in Table 2.

### 2.3. Grid Sensitivity and Model Verification

**Figure 4:**
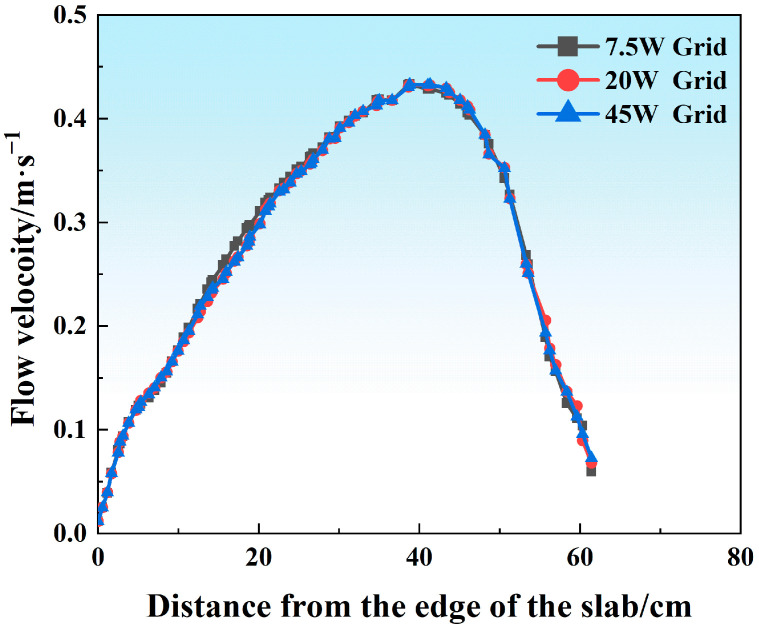
Comparison of the free surface velocity’s evolution using various grid densities (grid number units: 1W=10 thousand).

Hexa mesh and tetri mesh have been compared in convergence tests, and tetri mesh shows better performance and was used in this work. To further exclude the effect of the grid densities on the prediction accuracy of the Finite Element Method (FEM)model, various grid numbers (75,000, 200,000, 450,000) were generated in the CAD model (Figure 2), and the same model parameters were set in each case for further comparisons (1.0 m/min casting speed, SEN depth of 120 mm, port angle of 8 degrees (hereinafter abbreviated as deg)). The evolution of the free surface melt velocity was plotted, as shown in Figure 4. It could be found that with an increase in the grid number from 75,000 to 450,000, the flow field’s evolution tended to be independent of the grid number and identical. In this case, to improve the computation efficiency and maintain the accuracy, a total grid number of 75,000 was enough to describe the flow characteristics in the current FEM system, which was also beneficial in terms of accelerating the computational speed.

**Figure 5:**
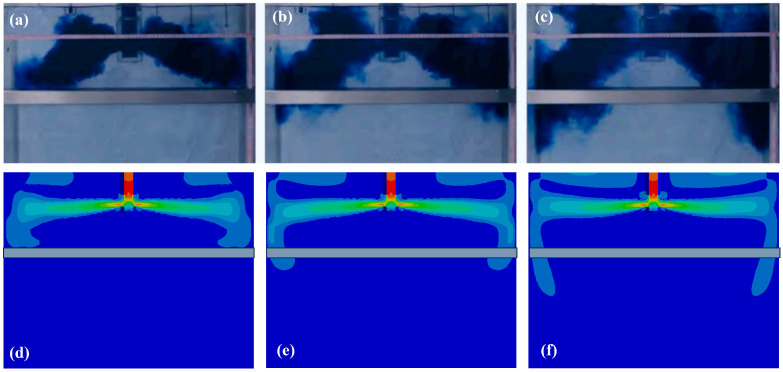
Comparison of the flow field evolution in the vertical cross-sectional direction of the mold between the water analogy experiment (**a**–**c**) and material modeling (**d**–**f**).

**Figure 6:**
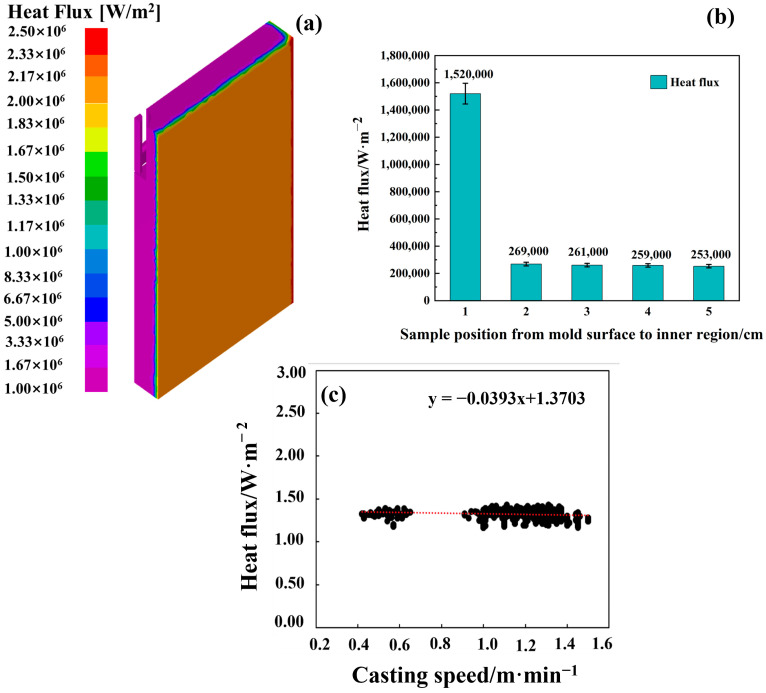
(**a**) The heat flux distribution in the mold region; (**b**) the average flux distribution in the mold region; (**c**) the industrial data from the caster.

Moreover, to further validate the suitability of the model for predicting the fluid flow pattern in the mold region for stainless steel, a benchmark water analogy experiment [30] was used to compare the results between mathematical modeling and an experiment, as shown in Figure 5. The results indicate an identical flow field distribution in both the modeling and experiment, especially at the mold’s free surface, which in turn proves the suitability of the FEM model for flow field prediction. Regarding thermal field prediction, the heat flux distribution (Figure 6a) in the mold region was simulated and compared with industrial data from the caster, as shown in Figure 6. The average flux distribution in the mold region fluctuated at around 1.5 MW/m^2^ (Figure 6b), which was very close to the data from the caster shown in Figure 6c.

## 3. Experiment Results

### 3.1. Morphological Characterization of Black Streak

**Figure 7:**
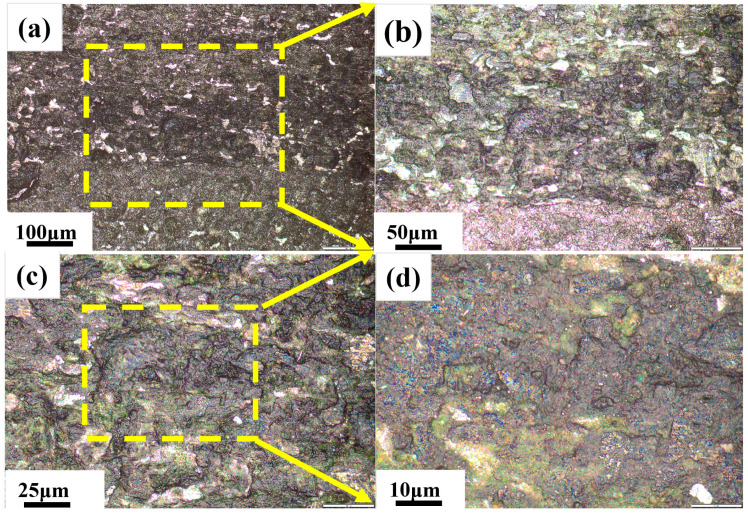
Macroscopic analysis of black streak: (**a**) 50× (**b**) 100×; (**c**) 200×; (**d**) 500×.

As is illustrated in Figure 1a, the black streak defect in the white-hot-rolled sheet sample was 428 mm in length and 1–7 mm in width. This defect presented as a rough longitudinal strip along the rolling direction with a heterogeneous chromatic distribution, displaying alternating dark-gray zones and zones with variable brightness intensities. The formation of heterogeneous zones primarily arose from the inherent inhomogeneity of the entrained mold flux itself and its changes during the entrainment and solidification processes. Figure 7a demonstrates the macroscopic morphology of the black streak under different metallographic microscopy conditions, revealing surface irregularities on the slab characterized by partial image blurring and poor focusing. Local magnification analysis (Figure 7d) identified black clusters on the hot-rolled plate surface, suggesting mold slag entering the molten steel during processing or iron oxide scale from surface oxidation during rolling.

**Figure 8:**
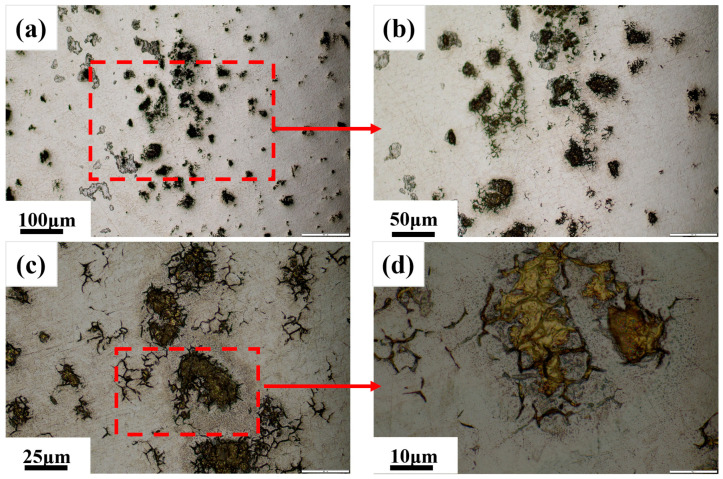
Micromorphology analysis of black streak: (**a**) 50×; (**b**) 100×; (**c**) 200×; (**d**) 500×.

Figure 8 displays the microstructural characteristics of the black streak observed through metallographic microscopy. At 50× dark substances were distinctly observed on the austenitic matrix surface. The enhanced resolution at 100× revealed an irregular surface topography with blocky particulates. A further examination at 500× confirmed the embedded nature of these dark constituents within the surface matrix. The combined analytical results indicate that the dark substances conclusively corresponded to mold slag. Moreover, the rough longitudinal strip (macro-defect) consisted of agglomerated slag clusters (micro-defects) with a variable composition, which is further confirmed in Section 3.2.

### 3.2. Black Streak Component Analysis

**Figure 9:**
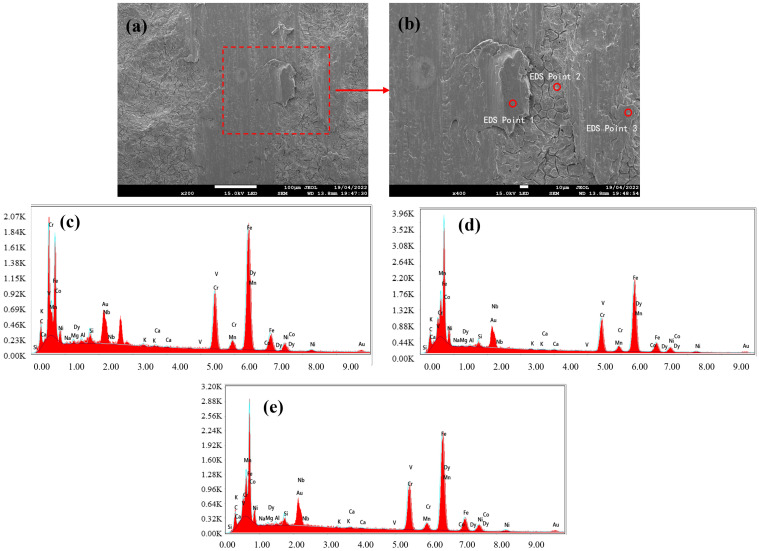
(**a**) SEM of sample 1; (**b**) enlarged area from (**a**), with EDS conducted at circled positions; (**c**) EDS element distribution map at point 1; (**d**) EDS element distribution map at point 2; (**e**) EDS element distribution map at point 3.

**Figure 10:**
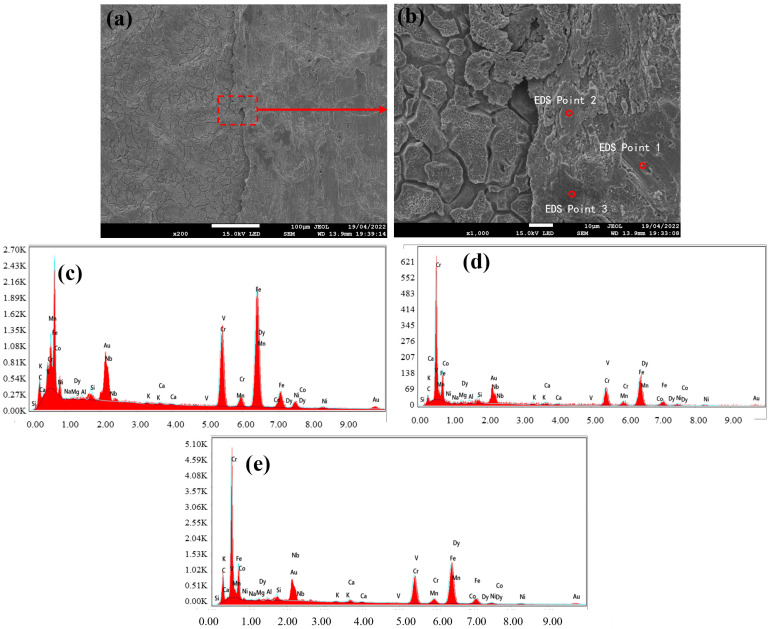
(**a**) SEM of sample 2; (**b**) enlarged area from (**a**), with EDS conducted at circled positions; (**c**) EDS element distribution map at point 1; (**d**) EDS element distribution map at point 2; (**e**) EDS element distribution map at point 3.

**Figure 11:**
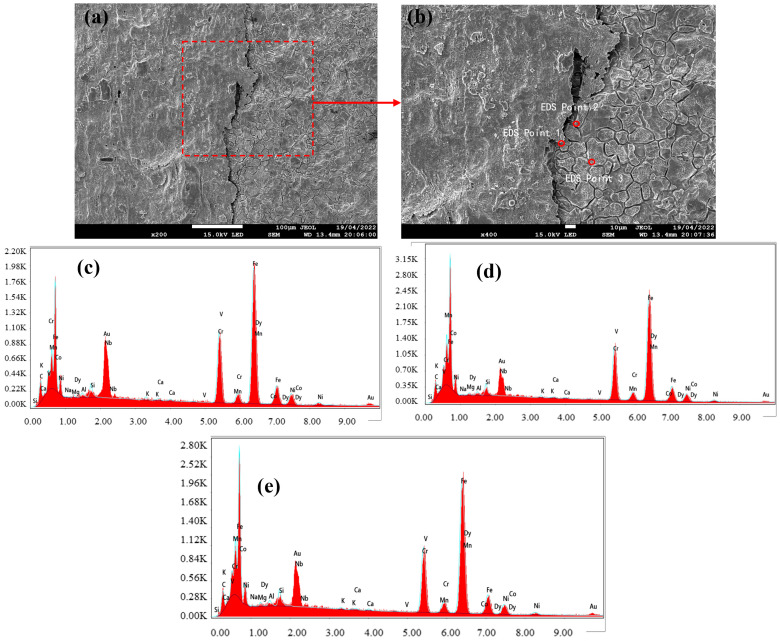
(**a**) SEM of sample 3; (**b**) enlarged area from (**a**), with EDS conducted at circled positions; (**c**) EDS element distribution map at point 1; (**d**) EDS element distribution map at point 2; (**e**) EDS element distribution map at point 3.

Following macroscopic and microscopic characterization, the samples subjected to metallographic microscopy were further analyzed via field emission scanning electron microscopy (SEM) coupled with energy-dispersive spectroscopy (EDS) to elucidate the elemental composition of the black streak defect. The EDS spot analysis results are presented in Figure 9, Figure 10 and Figure 11. The observations from SEM revealed that the defective region exhibited surface irregularities with distinct textural roughness. Notably, well-defined grain structures persisted in areas adjacent to the black streak, indicating no microstructural degradation in the peripheral regions. The quantitative elemental distributions across the three sampling locations are summarized in Table 3, Table 4 and Table 5, with consolidated EDS point analysis data provided in Table 6. A critical examination of Table 6 demonstrates the presence of Ca, Si, Al, Mg, Na, and K—characteristic components of continuous casting mold slag—in addition to matrix elements (Fe, Cr, Ni) inherent to the steel grade. This elemental signature conclusively confirms the direct correlation between black streak formation and the presence of mold slag during the continuous casting process.

## 4. Discussion

**Figure 12:**
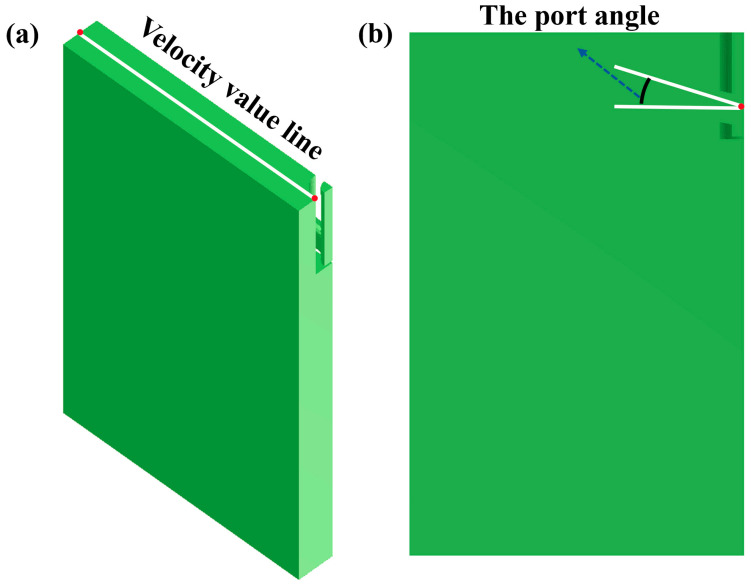
(**a**) Diagram of line along which velocity values were measured; (**b**) three-dimensional model of crystallizer.

Based on the analysis and existing research, the formation mechanism of black streaks in hot-rolled plates is intrinsically linked to the mold slag behavior during continuous casting. An elevated turbulence intensity at the steel meniscus triggers heterogeneous phase separation within the liquid flux layer under shear stress. This hydrodynamic instability enables flux droplets to breach the interfacial constraints and infiltrate the solidification front, constituting characteristic metallurgical slag entrapment. The entrapped slag particles propagate through the subsequent rolling, solution treatment, and pickling stages, evolving into coarse, black, linear surface defects after multi-stage processing. In addition, the critical surface velocity threshold for shear-induced slag entrapment was determined to be 0.35 m/s based on Equation (8) and Table 7 [31,32]. Process optimizations targeting the casting speed, the port angle of the SEN, and the depth of the SEN effectively suppressed the meniscus flow intensity, thereby reducing the probability of shear-induced slag entrapment. During the post-processing of the calculation results, a line was considered on the surface of the molten steel crystallizer, and the velocity distribution curve on this line was obtained according to the numerical calculation results. A diagram of the line along which the velocity values were measured is shown in Figure 12.

(8) $$\nu_{s,crit}=[1+C_{0}{\left(\frac{\rho_{s}}{\rho_{m}}\right)}^{\frac{2}{3}}{\left(\frac{\nu_{s}}{\nu_{m}}\right)}^{\frac{1}{3}}\cdot {\left[\frac{36\left(\rho_{m}-\rho_{s}\right)g\sigma_{ms}}{\rho_{s}^{2}}\right]}^{\frac{1}{4}}$$

### 4.1. Influence of Different Casting Speeds on Flow Velocity and Temperature in Mold and at Slag–Steel Interface

**Figure 13:**
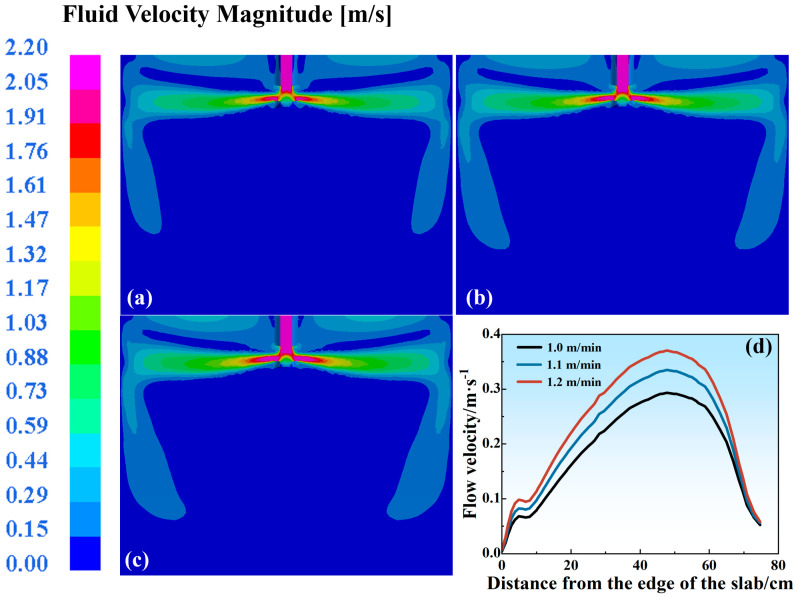
Flow velocity distribution in the mold at different casting speeds: (**a**) 1.0 m/min; (**b**) 1.1 m/min; (**c**) 1.2 m/min. (**d**) Liquid steel surface flow velocity.

During continuous casting, the casting speed critically governs slag entrainment phenomena within the mold. Under constant slab width conditions, a moderate elevation of the casting speed enhances productivity. However, excessive speeds amplify the molten steel fluctuations in narrow-face regions and at free surfaces, elevating the breakout risks and promoting shear-induced slag entrainment, ultimately destabilizing the casting processes and degrading the strand quality. Therefore, investigating the impact of varying casting speeds on the molten steel flow velocity becomes imperative [33]. Figure 13a–c present the flow velocity distributions under different casting speeds when the depth of the SEN was 130 mm and the port angle of the SEN was 8 deg. The molten steel develops dual recirculation zones (upper and lower) upon discharge from the SEN. An increased casting speed elevates the SEN jet’s impingement velocity, thereby augmenting the penetration depth. Concurrently, the expansion of the upper recirculation zone intensifies the meniscus fluctuation amplitudes, heightening the shear-induced slag entrainment probabilities. An enhanced impingement velocity against the narrow face exacerbates meniscus turbulence. This velocity-dependent flow evolution underscores the necessity for a dynamic equilibrium between productivity enhancement and flow stability during process optimization, mandating the prioritization of flow control in two critical domains: narrow-face impingement zones and meniscus regions.

Figure 13d illustrates variations in the liquid steel surface flow velocity under different casting speeds, with the curve peaks representing the maximum surface velocities. Under the current mold slag conditions, the critical meniscus velocity is determined to be 0.35 m/s. Exceeding this threshold indicates a heightened shear-induced slag entrainment susceptibility, while subcritical values correspond to reduced entrapment probabilities. At a 1.0 m/min casting speed, the peak surface velocity measures 0.29 m/s, remaining below the critical threshold. A casting speed elevation to 1.1 m/min yields a maximum velocity of 0.34 m/s, deviating by merely 0.01 m/s from the critical value. Notably, when the casting speed escalates to 1.2 m/min, the peak surface velocity surpasses the critical limit at 0.37 m/s. This condition elevates the shear-induced slag entrainment risks, subsequently increasing the scab defect probabilities during downstream rolling processes. Each 0.1 m/min casting speed increment induces a 0.03 m/s augmentation in the maximum meniscus velocity, correlating with progressive increases in both the shear-induced slag entrainment likelihood and black streak formation during rolling operations.

**Figure 14:**
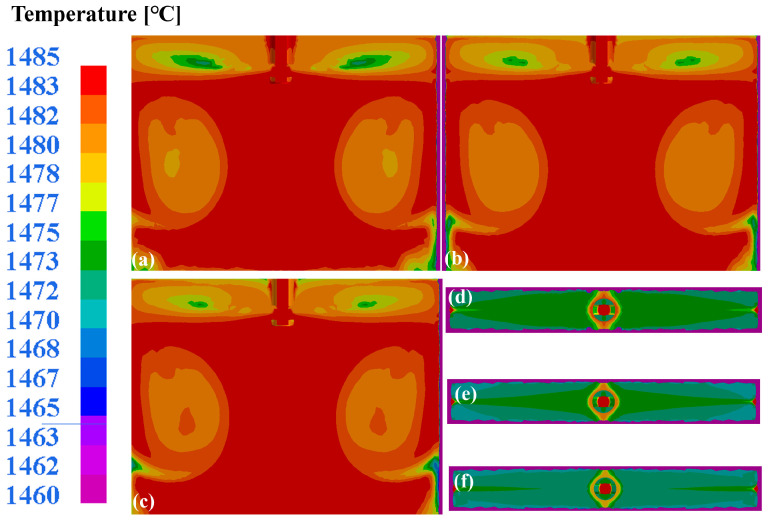
Wide-surface temperature distribution in mold at different casting speeds: (**a**) 1.0 m/min; (**b**) 1.1 m/min; (**c**) 1.2 m/min. Temperature distribution at slag–steel interface: (**d**) 1.0 m/min; (**e**) 1.1 m/min; (**f**) 1.2 m/min.

While preventing excessive meniscus flow velocities to mitigate shear-induced slag entrainment defects, it remains imperative to ensure the proper melting of mold slag at the steel surface. Incomplete flux melting may generate solidified flux clusters that become entrapped as inclusion defects. Furthermore, unmelted flux fails to effectively absorb nonmetallic inclusions in molten steel, leading to their retention in the strand and compromising the internal quality. Consequently, rapid flux melting at the mold surface critically governs the strand quality. Figure 14a–c presents the wide-face temperature distributions under varying casting speeds, revealing dual recirculation zones analogous to the flow field patterns. Increasing the casting speed elevates the average recirculation zone temperatures (Figure 14a–c), concurrently expanding the slag–steel interface temperature ranges (Figure 14d–f). At a 1.0 m/min casting speed, the slag–steel interface temperature spans 1458–1473 °C. Elevating the casting speed to 1.2 m/min raises this range to 1468–1478 °C, achieving a 10 °C average temperature increase that enhances the flux melting homogeneity. This improved fluidity facilitates uniform flux infiltration into the mold–strand gap, optimizing the lubrication effectiveness. However, excessive velocities intensify the meniscus and narrow-face flow fluctuations, increasing the probability of shear-induced slag entrainment. A comparative analysis confirmed that at a 1.0 m/min casting speed, the slag–steel interface temperature exceeds 1450 °C, ensuring the complete melting of the mold flux to meet operational requirements. Simultaneously, this casting speed minimizes the meniscus fluctuations, significantly reducing the probability of slag entrapment. Consequently, a 1.0 m/min casting speed represents the optimal casting speed within the scope of this study.

### 4.2. Influence of Different Port Angles of SEN on Flow Velocity and Temperature in Mold and at Slag–Steel Interface

**Figure 15:**
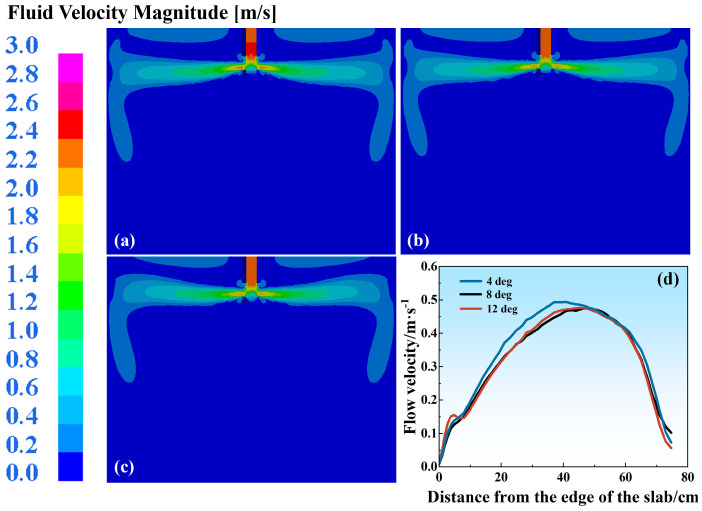
Flow velocity distribution in mold at different port angles of SEN: (**a**) 4 deg; (**b**) 8 deg; (**c**) 12 deg. (**d**) Liquid steel surface flow velocity.

During continuous casting operations, molten steel is delivered into the mold through the SEN, where nozzle geometric parameters such as the port angle critically govern the flow dynamics. Downward-tilted nozzles are widely preferred for high-speed casting due to their stability and minimized turbulence. However, the inherent properties of stainless steel make its production susceptible to metallurgical defects caused by inadequate mold flux melting. An upward-tilted nozzle orientation offers a distinct advantage by directing the flow toward the mold flux layer, significantly enhancing the thermal transfer and melting efficiency. Figure 15a–c present the molten steel flow velocity distributions under fixed casting conditions (1.0 m/min casting speed, 145 mm SEN depth) with different port angles of the SEN. Increasing the port angle reduces the jet penetration depth due to amplified upward-momentum components. Despite maintaining dual recirculation patterns, upward-tilted nozzles exhibit reduced upper recirculation zone dimensions and diminished meniscus fluctuation amplitudes, consequently lowering the shear-induced slag entrainment probabilities. Figure 15d quantifies the flow velocity variations across different port angles of the SEN, revealing maximum velocities of 0.291 m/s at 4 deg, 0.289 m/s at a port angle of 8 deg, and 0.238 m/s at 12 deg. In addition, all these values are subcritical with regard to the 0.35 m/s threshold. The maximum surface velocity decreases by 0.053 m/s compared to its value at 4 deg, a reduction of 18.2%. And the difference between 4 deg and 8 deg is negligible, with a decrease of only 0.002 m/s.

**Figure 16:**
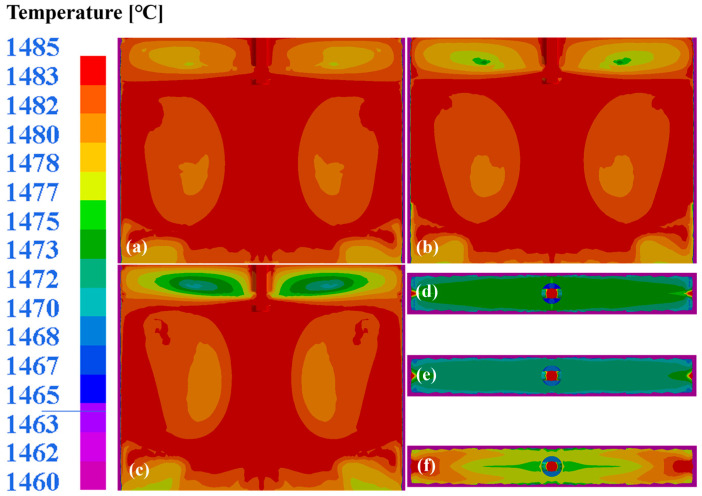
Wide-surface temperature distribution in mold at different port angles of SEN: (**a**) 4 deg; (**b**) 8 deg; (**c**) 12 deg. Temperature distribution at slag steel interface: (**d**) 4 deg; (**e**) 8 deg; (**f**) 12 deg.

Concurrently, sufficient slag–steel interface temperatures exceeding the flux melting points are essential to prevent quality degradation due to unmelted flux. Figure 16 illustrates the wide-face thermal profiles and interface temperatures under identical casting parameters. Increasing the tilt port angle from 4 deg to 12 deg reduces the upper recirculation zone’s core temperatures from 1478 °C to 1471 °C, a decrease of 7 °C, while intensifying vortex generation and the flow turbulence. The interface temperature range transitions from 1460–1472 °C to 1467–1490 °C with an improvement in the port angle from 4 deg to 12 deg. In addition, all the configurations satisfy the flux melting requirements. The configuration with 12 deg achieves the fastest flux melting initiation through an optimized thermal distribution, combined with minimized meniscus velocities (0.238 m/s). This synergistic effect between the thermal and hydrodynamic conditions establishes a port angle of 12 deg as optimal for defect minimization and product quality enhancement under the specified casting parameters.

### 4.3. Influence of Different Depths of SEN on Flow Velocity and Temperature in Mold and at Slag–Steel Interface

**Figure 17:**
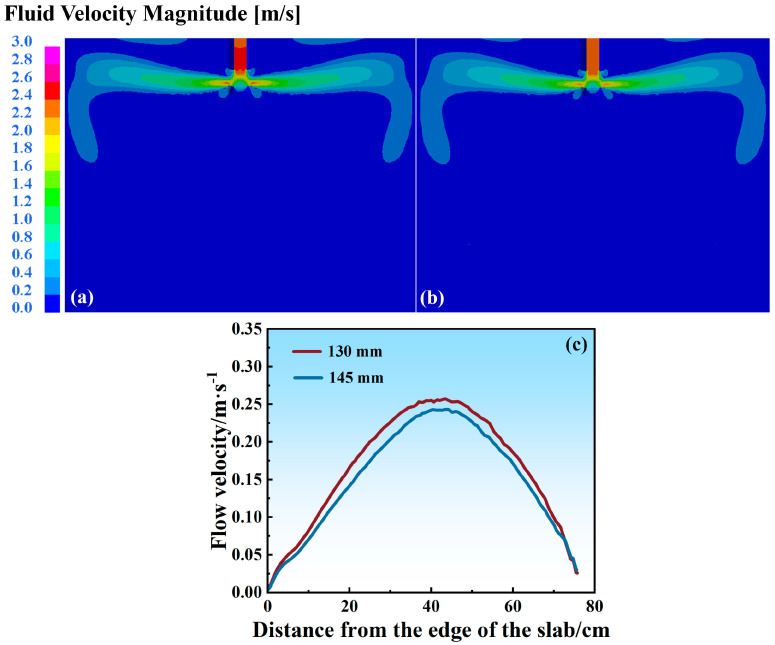
Flow velocity distribution in mold at different depths of SEN: (**a**) 130 mm; (**b**) 145 mm. (**c**) Liquid steel surface flow velocity.

The immersion depth of the SEN critically modulates the upper recirculation zone characteristics in continuous casting molds. Increasing the SEN depth expands the recirculation zone coverage, reducing the meniscus velocity amplitudes and stabilizing the surface fluctuations. However, excessive immersion diminishes the mold surface’s thermal flux density, impairing the mold slag’s melting efficiency and producing inadequate liquid slag layers that predispose the meniscus regions to shear-induced slag entrainment. Conversely, reduced SEN immersion results in contracted recirculation zones, amplifying the meniscus velocities and hydrodynamic disturbances, thereby elevating the shear-induced slag entrapment risks. Figure 17a–b compare the velocity distributions under fixed casting parameters (1.0 m/min casting speed, 12° nozzle angle) with 130mm and 145mm SEN immersion depths. The deeper 145 mm immersion enhances jet penetration while decreasing the size of the upper recirculation zones and reducing the surface fluctuations. Figure 17c quantifies the meniscus velocity profiles, revealing maximum velocities of 0.257 m/s (130 mm) and 0.243 m/s (145 mm), both subcritical with regard to the 0.35 m/s threshold. The 145 mm configuration demonstrates the lowest entrainment probability due to reduced shear stress at the slag–steel interfaces, where interfacial tension dominates over hydrodynamic forces.

**Figure 18:**
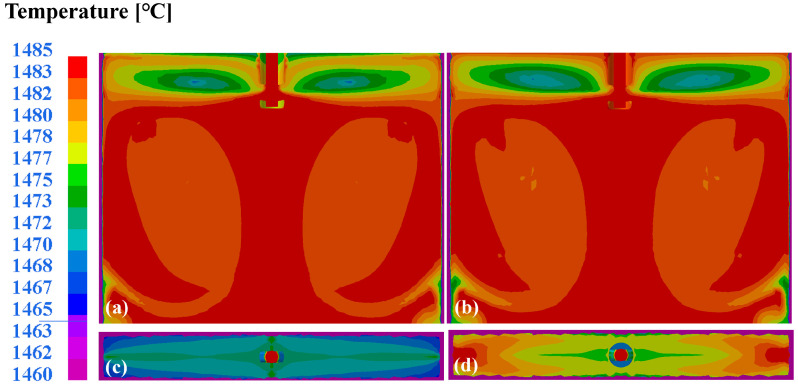
Wide-surface temperature distribution in mold at different depths of SEN: (**a**) 130 mm; (**b**) 145 mm. Temperature distribution at slag–steel interface: (**c**) 130 mm; (**d**) 145 mm.

The thermal analyses shown in Figure 18a–b demonstrate diminished vortex formation in the upper recirculation zones with deeper SEN immersion (145 mm vs 130 mm), corroborating the suppressed surface turbulence. The 130 mm immersion exhibits 3 °C lower core temperatures in the upper recirculation zones compared to the 145 mm configuration. Crucially, the slag–steel interface temperatures under a 145 mm immersion (1475–1485 °C) significantly exceed those at a 130 mm depth (Figure 18c–d). This is more beneficial to achieving the optimal flux melting conditions. These combined thermal–hydrodynamic effects establish 145 mm SEN immersion as superior for process stability: the reduced meniscus velocities mitigate the entrainment risks while the elevated interface temperatures ensure adequate flux lubrication. This dual optimization mechanism effectively balances the flow control and thermal management requirements in high-speed casting operations.

### 4.4. Optimization of Physical Properties of Mold Slag Based on Critical Surface Velocity

**Figure 19:**
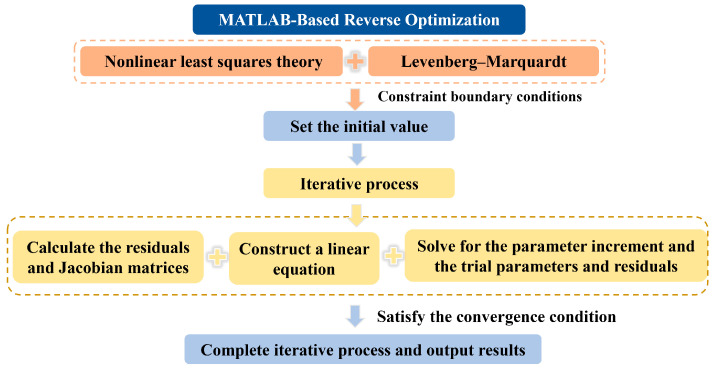
The specific calculation route for MATLAB-based reverse optimization.

Based on the nonlinear least squares theory, the conflicting equations in metallurgical processes are transformed into optimization and inverse problems. The optimal approximate solution is sought by minimizing the residual sum of squares, where the residuals represent the deviations between the model-predicted and experimentally measured values in metallurgical reactions. This study employs the Levenberg–Marquardt (LM) algorithm, a nonlinear least squares optimization method that integrates the advantages of the Gauss–Newton method and gradient descent. By introducing a damping factor to dynamically adjust the parameter update strategies during iteration, the algorithm enhances both the convergence efficiency and numerical stability for kinetic parameter identification in metallurgical multiphase reactions. For a given nonlinear constitutive model and n observation data points, ${\left\{(x_{i},y_{i}\right\}}_{i=1}^{n}$, the objective is to determine the parameter vector that minimizes the residual sum of squares, formulated based on the nonlinear mapping between the process parameters and system responses in metallurgical operations. The nonlinear constitutive model is given by Equations (9) and (10) is described as the residual sum of squares. Additionally, the specific calculation route is shown in Figure 19.

(9) y=f(x;θ)

(10) $$S\left(\theta \right)=\sum_{i=1}^{n}[{y_{i}-f(x_{i};\theta )]}^{2}$$

where x is the function argument and θ is the parameter vector. $x_{i}$ and $y_{i}$ are the observed values.

Based on the analysis above, the minimum value of the maximum surface flow velocity of molten steel was achieved at a casting speed of 1.0 m/min with an SEN depth of 145 mm and an SEN port angle of 12 deg. Two maximum surface velocity values (0.238 m/s and 0.243 m/s) were obtained under these optimized operational conditions, which were identified as the critical velocity for shear-induced slag entrapment, as defined by Equations (11) and (12). Through MATLAB-based reverse optimization algorithms, the optimal physical properties of mold slag were determined: a density of 2800 kg/m^3^, kinematic viscosity of 4.756×10^−6^ m^2^/s, and slag–steel interfacial tension of 0.01 N/m. Meanwhile, the residual sum of squares was 1.25×10^−5^, which demonstrates the accuracy of the reverse calculation optimization results.

(11) $$[1+C_{0}{\left(\frac{\rho_{s}}{\rho_{m}}\right)}^{\frac{2}{3}}{\left(\frac{\nu_{s}}{\nu_{m}}\right)}^{\frac{1}{3}}\cdot {\left[\frac{36\left(\rho_{m}-\rho_{s}\right)g\sigma_{ms}}{\rho_{s}^{2}}\right]}^{\frac{1}{4}}-0.238=0$$

(12) $$[1+C_{0}{\left(\frac{\rho_{s}}{\rho_{m}}\right)}^{\frac{2}{3}}{\left(\frac{\nu_{s}}{\nu_{m}}\right)}^{\frac{1}{3}}\cdot {\left[\frac{36\left(\rho_{m}-\rho_{s}\right)g\sigma_{ms}}{\rho_{s}^{2}}\right]}^{\frac{1}{4}}-0.243=0$$

where $\rho_{s}$, $\nu_{s}$, and $\sigma_{ms}$ are unknown and $\rho_{m\;}$ and $\nu_{m}$ are 7010 kg/m^3^ and 9.7 × 10^−7^ m^2^/s.

## 5. Conclusions

(1) Metallographic analysis using optical microscopy revealed black streak defects measuring 428 mm in length with widths ranging from 1 to 7 mm. SEM-EDS characterization confirmed the presence of mold slag components within defect regions, identifying shear-induced slag entrainment during continuous casting as the fundamental mechanism of their formation. The entrained slag particles evolved into elongated black streak defects through the subsequent processing stages, significantly compromising the surface quality and industrial profitability. This defect formation mechanism underscores the critical need for optimizing mold slag’s properties and flow control strategies in continuous casting operations.

(2) Numerical simulations revealed critical process parameter effects on the flow–thermal behavior. When the casting speed increased from 1.0 to 1.2 m/min, the value of the maximum surface velocity improved from 0.29 m/s to 0.37 m/s, an increase of 27.6%. And with the enhancement of the casting speeds, the slag–steel interface temperature improved by 10 °C. As the port angle of the SEN was enlarged from 4 deg to 12 deg, the surface velocity was reduced by 18.2% from 0.291m/s to 0.238 m/s. However, that increased the interface temperature to 1467–1490 °C. In addition, the surface velocity exhibited a reduction from 0.257 m/s to 0.243 m/s, decreasing by 0.014 m/s with an increase in the depth of the SEN from 135 mm to 145 mm. The optimal velocities minimizing the shear-induced slag entrainment probability were identified to be 0.238 and 0.243 m/s.

(3) Building upon the findings in (2) and the critical slag entrainment threshold determined using Equation (8), MATLAB-based reverse optimization inversely determined the optimal physical parameters for mold slag design: a density of 2800 kg/m^3^, kinematic viscosity of 4.756 × 10^−6^ m^2^/s, and slag–steel interfacial tension of 0.01 N/m. These reverse-engineered parameters establish theoretical foundations for developing high-performance mold slag that simultaneously suppresses slag entrainment while maintaining essential lubrication and thermal regulation functions in stainless steel continuous casting operations.

## Figures and Tables

**Table 1:** Main chemical composition of AISI 304 stainless steel.

C	Si	Mn	S	C	Ni	Mo	Cu	N	Fe
0.045	0.45	1.25	≤0.006	18.00	8.00	≤0.50	≤0.50	0.04	Bal.

**Table 2:** Simulation parameters used in the modeling.

NSTEP	TFINAL	TSTOP	DT	DTMAXFILL	DTMAX
2000	45 s	1411.3 °C	0.001 s	0.1 s	0.1 s

Note: NSTEP is the number of timesteps in the simulation, TFINAL is the final amount of time taken for the whole simulation, TSTOP is the final temperature required for simulation termination, DT is the initial timestep size, DTMAXFILL is the maximum timestep in the flow simulation, and DT MAX is the maximum timestep in the thermal simulation.

**Table 3:** EDS results for sample 1.

Element	Fe	Cr	Ni	O	Si	Mn	Al	Ca	Mg	Na	K
EDS #1	65.56	6.71	5.73	18.27	1.11	0.42	0.46	0.20	0.93	0.34	0.27
EDS #2	68.86	19.37	7.94	1.69	1.05	0.95	0.34	0.18	0.20	0.09	0.30
EDS #3	67.90	19.80	7.47	1.14	1.17	0.87	0.39	0.32	0.36	0.20	0.38
Average	67.12	15.29	7.05	7.03	1.11	0.75	0.40	0.23	0.50	0.21	0.32

**Table 4:** EDS results for sample 2.

Element	Fe	Cr	Ni	O	Si	Mn	Al	Ca	Mg	Na	K
EDS #1	64.38	26.12	5.67	1.14	0.93	0.48	0.35	0.28	0.24	0.10	0.31
EDS #2	46.49	17.96	5.17	21.33	1.51	2.90	0.80	1.37	0.90	0.41	1.15
EDS #3	52.64	22.12	2.87	17.23	1.17	1.51	0.56	1.07	0.44	0.05	0.33
Average	54.50	22.07	4.57	13.23	1.20	1.63	0.57	0.91	0.53	0.19	0.60

**Table 5:** EDS results for sample 3.

Element	Fe	Cr	Ni	O	Si	Mn	Al	Ca	Mg	Na	K
EDS #1	67.71	20.17	7.76	1.12	1.08	0.95	0.35	0.18	0.27	0.13	0.29
EDS #2	66.89	20.88	8.80	0.80	0.94	0.49	0.33	0.24	0.19	0.07	0.36
EDS #3	67.68	19.51	8.04	0.99	1.17	1.25	0.45	0.18	0.37	0.18	0.19
Average	67.43	20.19	8.20	0.97	1.06	0.90	0.38	0.20	0.28	0.13	0.28

**Table 6:** Statistical table of main elemental content in black streak area.

Sample No.	Fe	Cr	Ni	O	Si	Mn	Al	Ca	Mg	Na	K
1	67.12	15.29	7.05	7.03	1.11	0.75	0.40	0.23	0.50	0.21	0.32
2	54.50	22.07	4.57	13.23	1.20	1.63	0.57	0.91	0.53	0.19	0.60
3	67.43	20.19	8.20	0.97	1.06	0.90	0.38	0.20	0.28	0.13	0.28

**Table 7:** The physical properties associated with each letter in Equation (8).

Symbol	Physical Property	Value
$C_{0}$	Constant	0.20
$\rho_{s}$	Slag density	3300 kg/m^3^
$\rho_{m}$	Density of molten steel	7010 kg/m^3^
$\nu_{s}$	Kinematic viscosity of the slag	2.0 × 10^−5^ m^2^/s
$\nu_{m}$	Kinematic viscosity of molten steel	9.7 × 10^−7^ m^2^/s
g	Gravitational acceleration	9.8 m^2^/s
$\sigma_{ms}$	Interfacial tension of steel slag	0.02 N/m

## Data Availability

The original contributions presented in this study are included in the article. Further inquiries can be directed to the corresponding author.
